# Supplementary material for: A lanthanide MOF with nanostructured node disorder
Source: Nat Commun. 2025 Apr 3;16:3209. doi: 10.1038/s41467-025-58402-4 (PMC11968993; doi:10.1038/s41467-025-58402-4)
Supplement: Supplementary file 1 — Supplementary information [file 41467_2025_58402_MOESM1_ESM.pdf]

Supplementary Information for:

A lanthanide MOF with nanostructured node disorder

Sarah L. Griffin, Emily G. Meekel, Johnathan M. Bulled, Stefano Canossa, Alexander Wahrhaftig-Lewis, Ella M. Schmidt, and Neil R. Champness*

Supporting Information Table of Contents

Experimental Details including Synthesis Page 2

Details of X-ray Crystallographic Experiments Page 6

Details of Diffuse Scattering Measurement and Analysis Page 19

Details of Computational Studies Page 20

References Page 29

Synthesis

Materials

Methyl 4-formylbenzoate, thiamine hydrochloride, hydrazine monohydrate, pyridine-2,6-dicarbonitrile, and all other solvents, bases and acids used were all purchased from either Sigma Aldrich, ThermoFisher, Alfa Aesar, or Fluorochem and were used without further purification.

Characterization

NMR spectra were recorded on a Bruker AVANCE NEO 400 MHz spectrometer and referenced to residual solvent peaks, unless otherwise stated. Deuterated solvents were used as specified. Single crystal XRD data were collected using a Rigaku Synergy-S dual source with a PhotonJet-S X-ray source and a HyPix-6000 detector. Data were collected at 100 K or 200 K through the use of an Oxford Cryosystems cryostream device.

Linker Synthesis

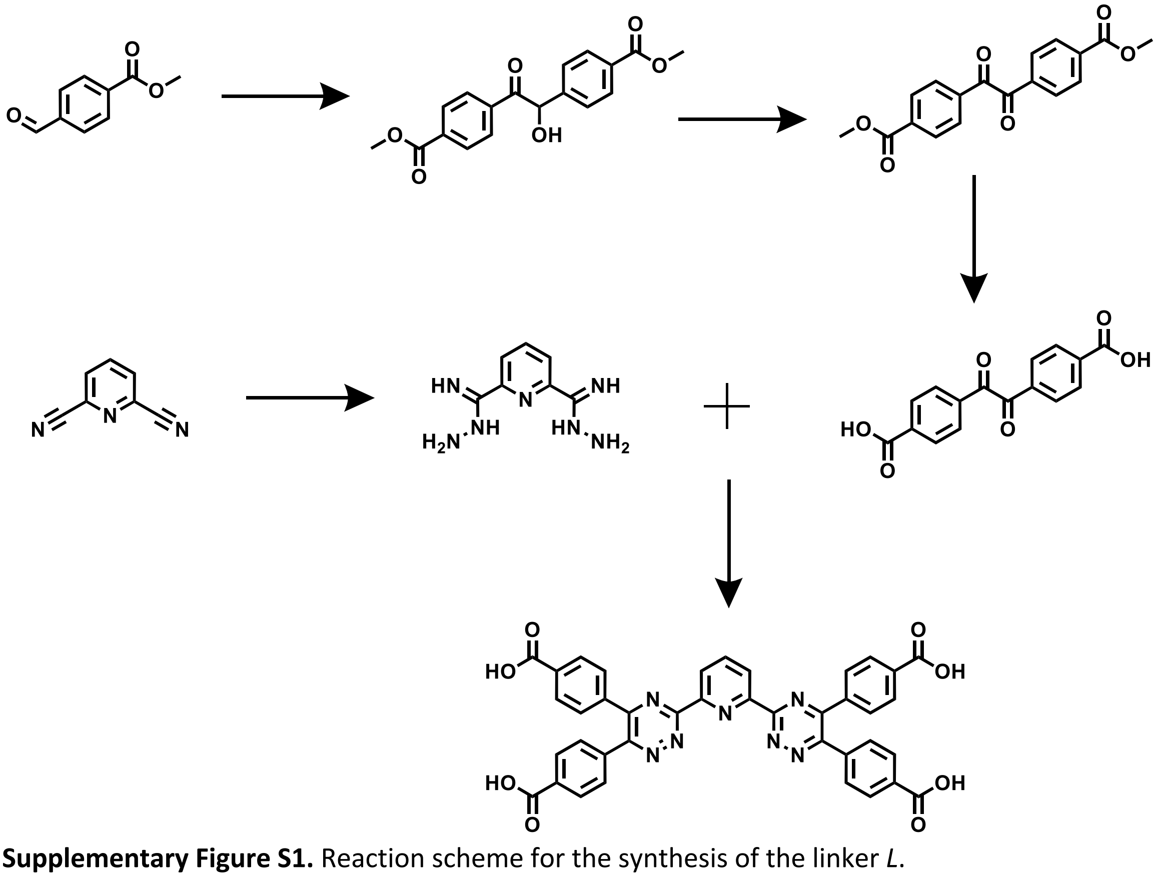

Supplementary Figure S1. Reaction scheme for the synthesis of the linker *L*.

Synthesis of 4,4'-(1-hydroxy-2-oxoethane-1,2-diyl)dibenzoate

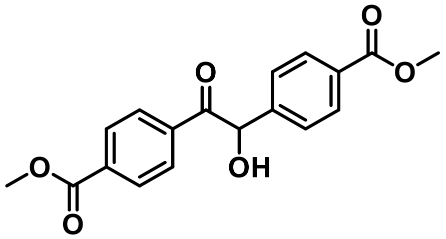

Thiamine hydrochloride (1.8 g, 5.34 mmol, 17 eq) was dissolved in water (6 mL) and methanol (18 mL) at 0 °C. A sodium hydroxide solution (2 M, 5 mL) was slowly added, followed by methyl 4-formylbenzoate (14.9 g, 90.8 mmol, 1 eq). The solution was slowly heated to 80 °C and left to stir for 2 hours, during which time a large quantity of solid precipitated. Upon completion the reaction was cooled and the solid filtered, washing with water, methanol and diethyl ether to give 4,4'-(1-hydroxy-2-oxoethane-1,2-diyl)dibenzoate as an off-white solid (10.521 g, 32.0 mmol, 71 %).

^{1}H NMR (400 MHz, $CDCl_3$) δ (ppm): 8.04 (dt, 2H), 7.99 (dt, 2H), 7.93 (dt, 2H), 7.39 (dt, 2H), 6.02 (d, 1H), 4.53 (d, 1H), 3.91 (s, 3H), 3.87 (s, 3H); ^{13}C NMR (400 MHz, $CDCl_3$) δ (ppm): 198.25, 166.39, 165.76, 143.00, 136.59, 134.75, 130.53, 130.46, 129.91, 128.94, 127.77, 76.21, 52.58, 52.23. Anal. Calcd. for $C_{18}H_{16}O_6$: C, 65.85; H, 4.91. Found: C, 65.93; H, 4.87. HRMS (ESI) calculated for $C_{18}H_{15}O_6$ $(M-H)^-$ 327.087, found m/z 327.086.

Synthesis of dimethyl 4,4'-oxalyldibenzoate

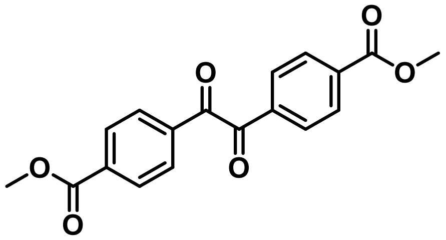

Dimethyl 4,4'-(1-hydroxy-2-oxoethane-1,2-diyl)dibenzoate (3.28 g, 10 mmol) was dissolved in dimethylsulfoxide (25 mL). Hydrobromic acid (40 % in water, 4.7 mL) was slowly added, and the mixture stirred at 55 °C for 24 hrs. Upon cooling, water was added to the reaction mixture before filtering off the solid. The solid was washed with copious amounts of water, followed by small amounts of methanol and diethyl ether. Dimethyl 4,4'-oxalyldibenzoate was collected as a pale-yellow solid (2.68 g, 15.3 mmol, 88 %).

^{1}H NMR (400 MHz, $CDCl_3$) δ (ppm): 8.18 (dt, 4H), 8.05 (dt, 4H), 3.96 (s, 6H); ^{13}C NMR (400 MHz, $CDCl_3$) δ (ppm): 192.89, 165.83, 135.76, 1.5.56, 130.18, 129.89, 52.68. Anal. Calcd. for $C_{18}H_{14}O_6$: C, 66.26; H, 4.32. Found: C, 66.44; H, 4.26. HRMS (ESI) calculated for $C_{18}H_{13}O_6$ $(M-H)^-$ 325.071, found m/z 325.186.

Synthesis of 4,4'-oxalyldibenzoic acid

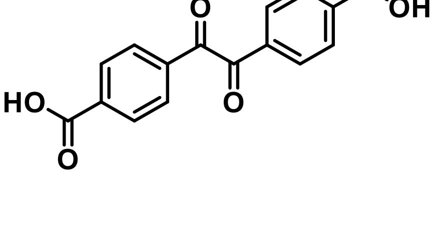

Dimethyl 4,4'-oxalyldibenzoate (2.63 g, 8.06mmol) was dissolved in acetic acid (200 ml). A solution of sulphuric acid (72 ml) and water (18 ml) were slowly added, and the mixture stirred at 110 °C overnight. Upon completion the reaction was cooled before filtering and washing with copious amounts of water. The solid was dried in an oven overnight at 50 °C, producing 4,4'-oxalyldibenzoic acid as yellow powder (1.68 g, 5.63 mmol, 70%).

^{1}H NMR (400 MHz, DMSO-d$_6$) δ (ppm): 13.46 (s, br), 8.14 (d, 4H), 8.06 (d, 4H); ^{13}C NMR (400 MHz, DMSO-d$_6$) δ (ppm): 193.84, 166.79, 136.92, 135.48, 130.56, 130.54. Anal. Calcd. for $C_{16}H_{10}O_6$: C, 64.43; H, 3.38. Found: C, 64.25; H, 3.37. HRMS (ESI) calculated for $C_{16}H_9O_6$ (M-H)$^-$ 297.040, found m/z 297.043.

Synthesis of pyridine-2,6-bis(carboximidhydrazide)

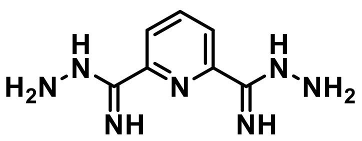

Pyridine-2,6-dicarbonitrile (2.00 g, 15.5 mmol, 1 eq) was dissolved in ethanol (50 mL) and stirred for 10 minutes. Hydrazine monohydrate (15 ml, 309 mmol, 20 eq) was slowly added, and the mixture stirred overnight at room temperature. Upon completion the reaction mixture was filtered and the solid washed with ethanol to give pyridine-2,6-bis(carboximidhydrazide) as a white solid (2.30 g, 11.9 mmol, 77 %).

^{1}H NMR (400 MHz, DMSO-d$_6$) δ (ppm): 7.80 (d, 2H), 7.64 (m, 1H), 6.04 (s, 4H), 5.24 (s, broad, integral 2H due to proton exchange); ^{13}C NMR (400 MHz, DMSO-d$_6$) δ (ppm) 150.78, 144.08, 136.46, 118.44. Anal. Calcd. for $C_7H_{11}N_7$: C, 43.51; H, 5.74; N, 50.75. Found: C, 43.57; H, 5.91; N, 54.74. HRMS (ESI) calculated for $C_7H_{11}N_7$ (M+CH$_3$OH+H)$^+$ 226.142, found m/z 226.951.

Synthesis of L

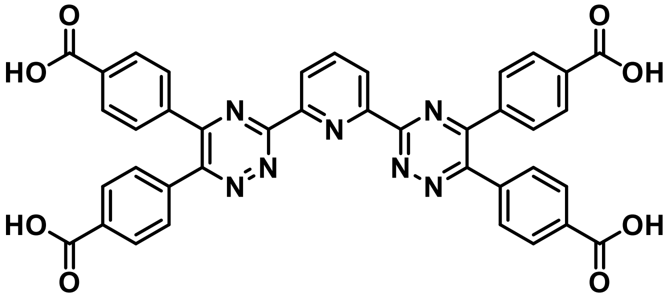

Pyridine-2,6-bis(carboximidhydrazide) (0.68 g, 3.52 mmol, 1 eq) and 4,4'-oxalyldibenzoic acid (2.19 g, 7.34 mmol, 2.1 eq) were stirred in dimethylformamide (25 mL) overnight at 80 °C. Upon cooling, methanol was added to the mixture to further precipitate solid, which was filtered and dried to give a yellow powder (1.58 g, 63%).

^{1}H NMR (400 MHz, DMSO-d$_6$) δ (ppm): 13.27 (s, 4H, br), 8.78 (d, 2H), 8.40 (t, 1H), 8.01 - 7.96 (m, 8H), 7.77 – 7.72 (m, 8H); ^{13}C NMR (400 MHz, DMSO-d^6) δ (ppm): 167.36, 167.22, 161.01, 156.49, 156.03, 153.52, 139.62, 139.55, 133.30, 132.41, 130.62, 130.31, 129.93, 129.77, 126.50. Anal. Calcd. for $C_{39}H_{23}O_8N_7$: C, 65.27; H, 3.23; N, 13.66. Found: C, 60.08; H, 3.57; N, 13.91. HRMS (ESI) calculated for $C_{39}H_{24}O_8N_7$ (M+H)$^+$ 718.169, found m/z 718.170.

Single crystals of *L* were grown following the heating of *L* in DMF at 120 °C for 24 hrs. The solution was then cooled and left for 4 weeks undisturbed, during which time yellow crystals grew.

Crystal data for linker: $C_{39}H_{21}N_7O_8 \cdot 2(C_3H_7NO) \cdot 2(C_2H_8N) \cdot 3.5[C_3H_7NO]$, M_r = 1209.84, crystal dimensions 0.25 x 0.14 x 0.06 mm, Triclinic, a = 9.3031 (1) Å, b = 18.0542 (2) Å, c = 21.4734 (2) Å, α = 113.642 (1) °, β = 93.090 (1) °, γ = 100.052 (1) °, V = 3222.41 (6) Å^3, T = 100 K, space group $P\bar{1}$, Z = 2, 113451 measured reflections, 12954 unique (R_{int} = 0.033) which were used in all calculations. The final R_1 = 0.084 for 11313 observed data $R[F^2 > 2\sigma(F^2)]$ and $wR(F^2)$ = 0.266. A solvent mask was calculated, and 138 electrons were found in a volume of 1055 A^3 in 1 void per unit cell. This is consistent with the presence of $3.5[C_3H_7NO]$ per asymmetric unit. Crystal structure data are available from the CCDC, deposition number 2370912.

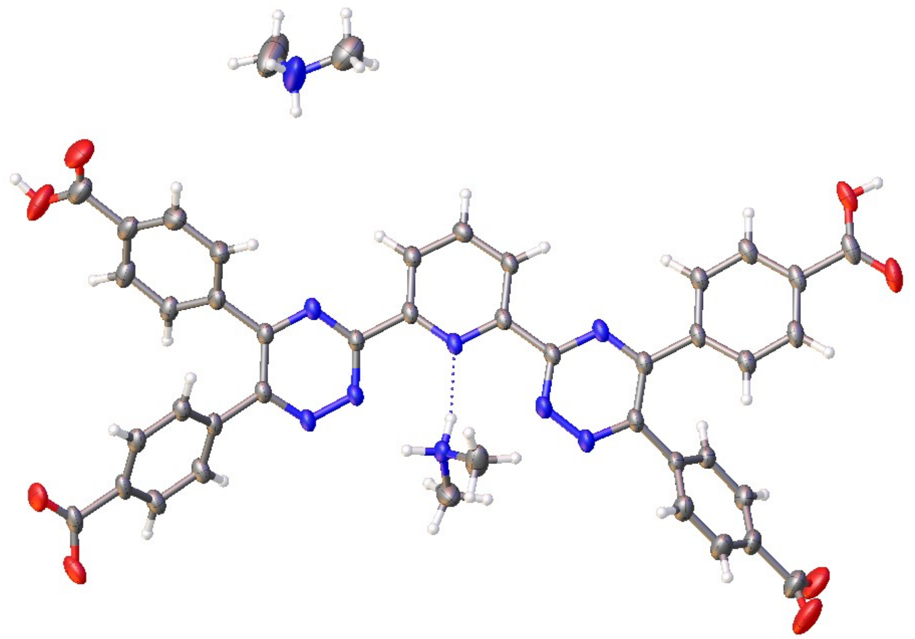

Supplementary Figure S2. View of the crystal structure of the linker used in the synthesis of UoB-100(Dy).

Synthesis of UoB-100(Dy)

Synthesis was adapted from a literature procedure.[S1] Linker *L* (7.0 mg, 9 µmol), dysprosium (III) nitrate hexahydrate (13.0 mg, 28 µmol), and 2-fluorobenzoic acid (0.60 g, 1.4 mmol) were added to a 25 ml Schott bottle, followed by *N*,*N*'-dimethylformamide (3 ml). The reaction jar was sonicated until all solids fully dissolved, followed by the addition of acetic acid (0.7 mL). The bottle was once again sonicated prior to placing in the oven at 120 °C for six days. During this period, yellow hexagonal crystals were formed. Upon cooling the crystals were isolated and washed with *N*,*N*'-dimethylformamide.

Single Crystal X-ray Diffraction Experiments

Average Structure Determination and Refinement

Single-crystal diffraction data for average structure determination were collected on a Rigaku XtaLAB Synergy-S X-ray diffractometer equipped with a HyPix-6000 hybrid photon counting detector and a Mo microfocus source. A suitable crystal was isolated and mounted on a MiTeGen loop in a droplet of a perfluoropolyether oil.

A series of omega scans were conducted (calculated by the automatic procedure from the software manufacturer) to ensure sufficient redundance and completeness of reflections, using an oscillation of 0.5° per frame. During data collection, the temperature was controlled by an Oxford CryoStream at a value of 250(1) K (reported by the Software interface). Data reduction was conducted by using the software CrysAlisPro.[S2] A clean, single lattice was identified from the harvested peaks. While no twinning/multicrystal was observed, systematically weaker superstructure reflections were spotted (Figure S3), and excluded from the integration as assigned to local structure effects. Intensities integration was based on a primitive hexagonal unit cell, while absorption correction was conducted by a multiscan approach as implemented in CrysAlisPro.

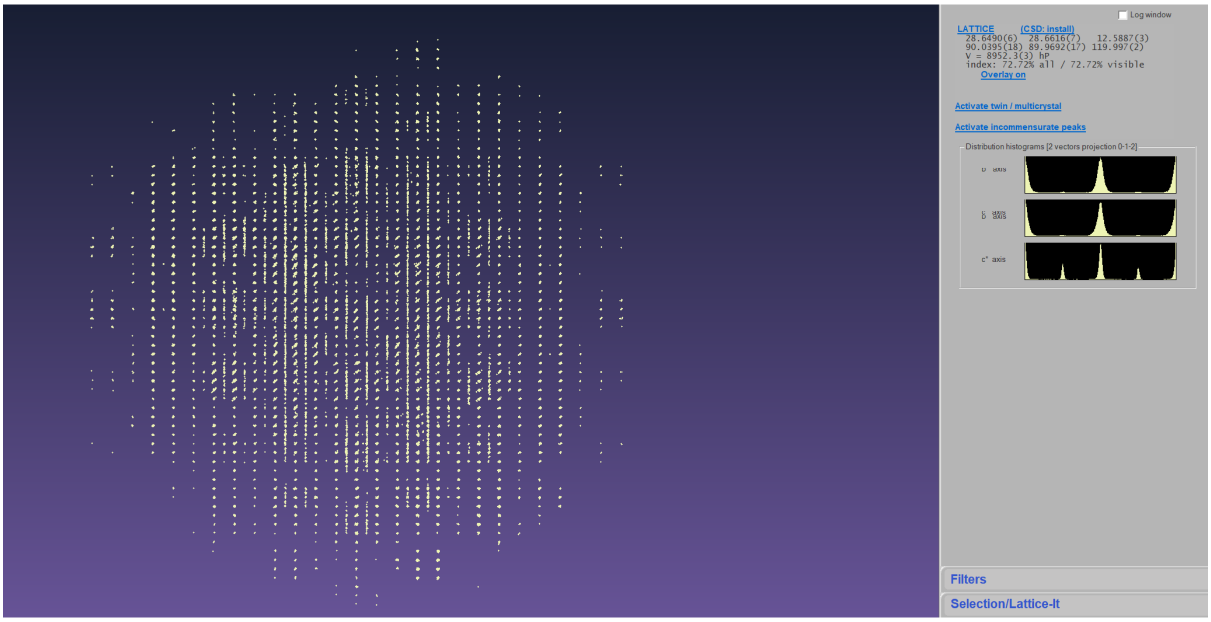

Supplementary Figure S3. Oriented view of the peaktable in CrysAlisPro, showing the presence of superstructure peaks along *c* direction.

Crystal structure solution and refinement was conducted by using the Olex2 software ver. 1.5.[S3] Structure solution was readily found by the program ShelXT[S4] used within the Olex2 user interface, already revealing the split positions of the Dy cluster and the coordinating parts of the linkers. The remaining atoms were assigned manually and a series of ShelXL restraints were used to drive the structure towards a reasonable molecular geometry, which also agreed with the underlying electron density (Figures S4, S5). The final refinement was conducted by the least square algorithm of the ShelXL structure refinement program.[S5] All hydrogens were fixed in idealized positions and not localized directly from the electron density residues.

Solvent masking was conducted to neglect the influence, on the refinements, of minor residual densities that were not possible to assign with adequate confidence. The mask was created by the dedicated function of the software Olex2, which masked a void of 6020.6 Å^3 per unit cell (67.4%), containing 1652 electrons belonging to possibly various kinds of solvent or modulator molecules.

See Table S1 for final refinement parameters.

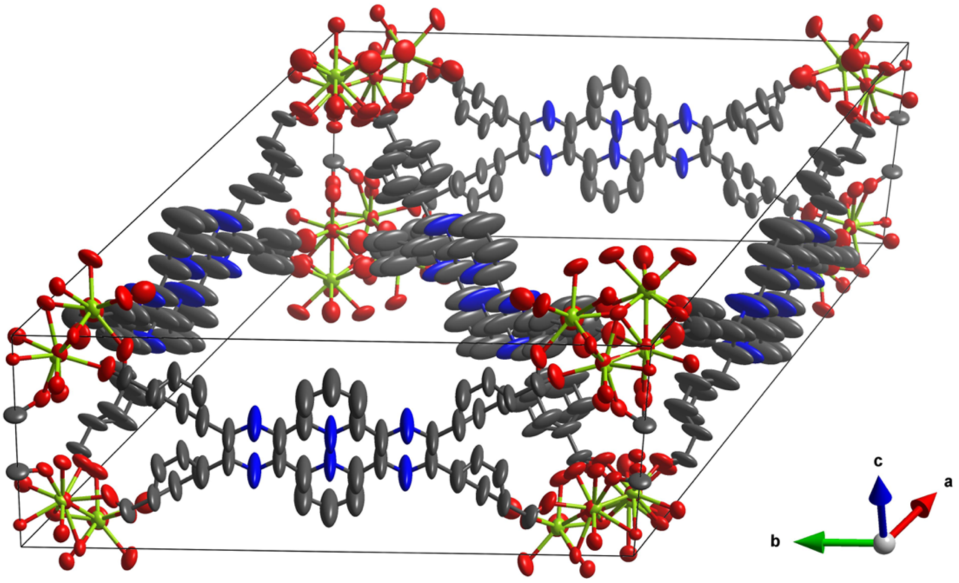

Supplementary Figure S4. Average structure modelled by SCXRD data (entire unit cell). Colour scheme: yellow-green: Dy, blue: N, dark grey: C, red: O. The hydrogen atoms are omitted for better clarity.

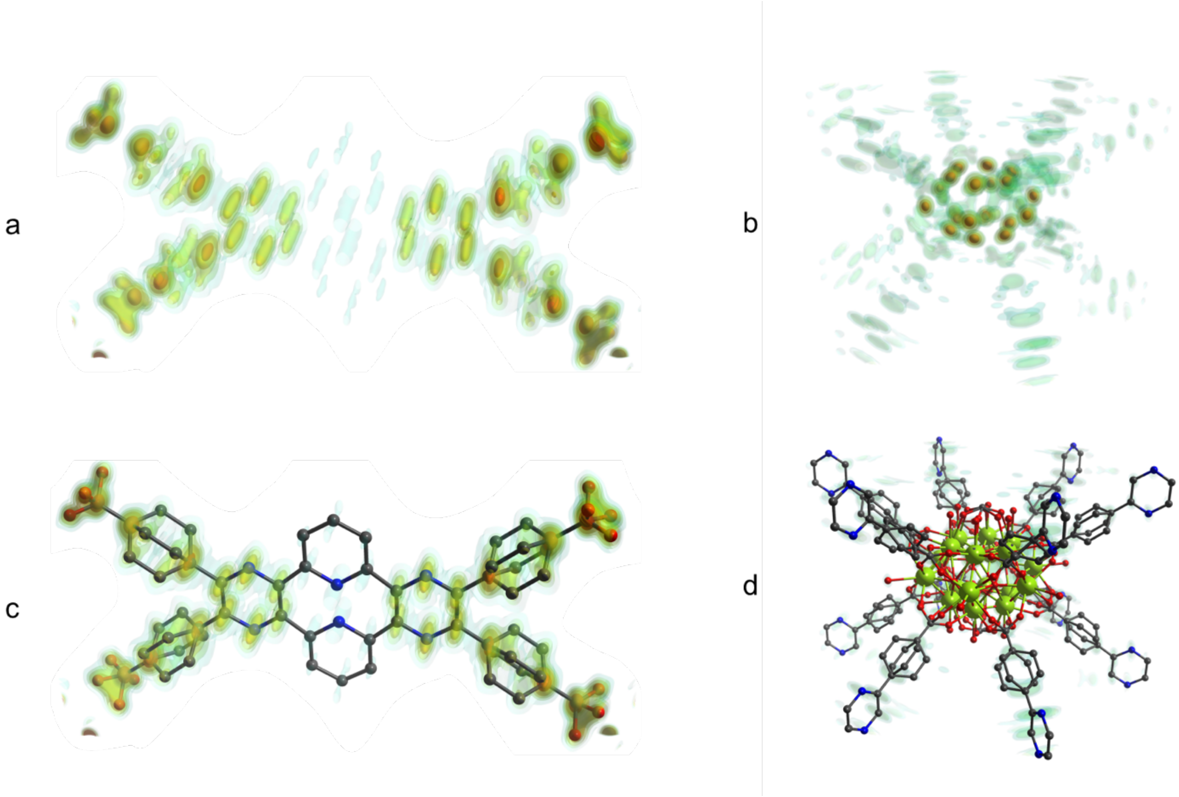

Supplementary Figure S5. View of the electron density observed in the regions occupied by the clusters and linkers (a, b) and respective overlaps with the modelled structure (c, d). Electron densities were calculated with the Function "Fourier synthesis" of the software Vesta, using the structure factors file generated by ShelXL with the command list3. Arbitrary isosurface levels have been used to enhance clarity.

Remarks on cluster modelling

While the atomic positions surrounding the 9 Dy atoms of the cluster are unambiguous, their assignment as either oxygens, hydroxy groups or water molecules were not. Based on purely geometrical considerations, there are 12 μ-3 bridging oxygens between Dy triplets. Those pointing above and below the cluster feature a distinctively smaller Dy-O-Dy angle, and have been therefore assigned as OH^- groups. The other ones, with shorter coordination distances (versus 2.3856(16) and 2.1382(15) Å compared to 2.542(5) Å) and nearly co-planar with their coordinated Dy centres, were assigned as O^{2-} species. Additional atomic positions with a larger coordination distance are present in the cluster's plane. These have been modelled as disordered water molecules based on their bonding distance of 2.57906(3) Å and the absence of other proximal atoms, which would be expected in case these were organic solvent molecules. There are additional positions above and below the cluster, which have been assigned as coordinated formate species and more water molecules. However, the 6OH+6O μ-3 groups, together with the 12 carboxylates provided by the surrounding linkers, already exceed considerably the charge balance with the 9 Dy atoms (30- versus 27+), without considering two atoms present inside the cluster, which could be O, OH, or a mixture of these species. To address

the mismatch of charges, we suggest that cations might be located between the clusters in a disordered fashion, along the c axis. It is however not possible to identify and model these species with the available chemical and crystallographic data.

As a last remark, we cannot completely exclude that part of the μ-3 oxygens are fluorides resulting from the decomposition of the fluorinated modulator, although these would be a minor fraction of the average cluster composition. A similar observation has been made for Ho-based MOFs prepared using a 2-fluorobenzoic acid modulator.[S6] The presence of fluorine in the MOF sample was confirmed by SEM-EDX microscopy. ^{19}F NMR spectroscopy was used to analyse both the supernatant solution and a dried MOF sample following digestion with deuterated sulphuric acid. The ^{19}F NMR spectrum for the supernatant shows the presence of 2-fluorobenzoic acid and 2-fluorobenzoate, with peaks at -112.85 and -118.76 ppm respectively. The ^{19}F spectrum for the acid digested MOF sample however has a single peak at 37.74 ppm, indicating the fluorine present in the MOF sample is not due to encapsulated 2-fluorobenzoic acid, and is more likely coordinated fluoride. Whilst these techniques confirm the presence of fluorine, they do not accurately quantify how much is present.

Supplementary Table S1. Summary of the SEM-EDX results.

Element	Line	Area (counts)	Weight %	Weight % σ	Atomic %
Carbon	k_series	15599	62.102	0.792	78.918
Oxygen	k_series	2092	15.291	0.586	14.588
Fluorine	k_series	948	6.160	0.452	4.949
Dysprosium	l_series	2342	16.446	0.723	1.545

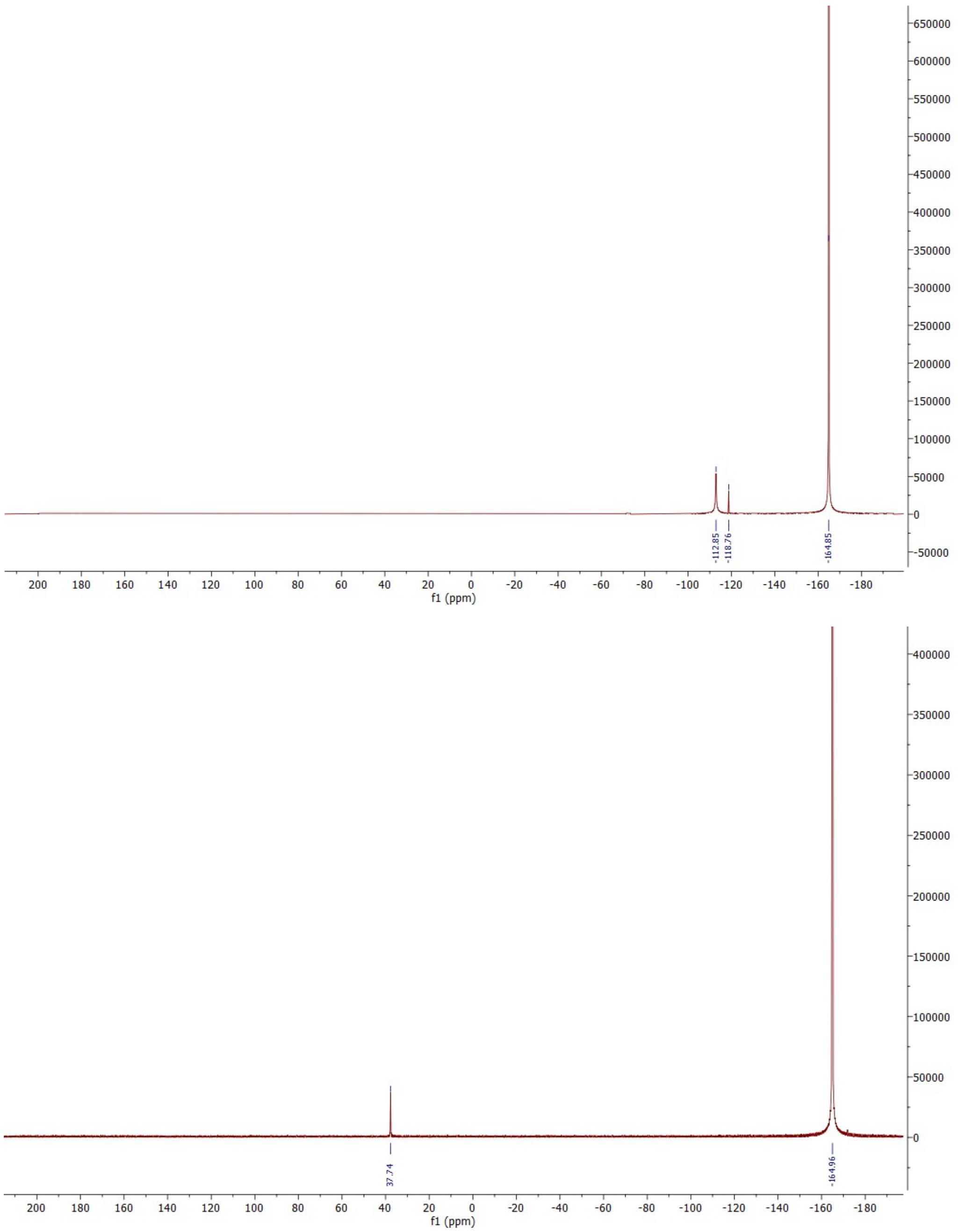

Supplementary Figure S6. ^{19}F NMR spectra of the reaction supernatant (top) and the acid digested MOF sample (bottom). Both samples were collected using DMSO-d_6 with hexafluorobenzene used as a fluorine reference (-164.9 ppm).

Supplementary Table S2. Crystal data and structure refinement.

Empirical formula	$C_{124}H_{97.96}Dy_9N_{15}O_{50}$
Formula weight	4060.63
Temperature/K	249.99(10)
Crystal system	hexagonal
Space group	P6/mmm
a/Å	28.6350(5)
b/Å	28.6350(5)
c/Å	12.5830(2)
α/°	90
β/°	90
γ/°	120
Volume/Å^3	8935.3(3)
Z	1
ρ_{calc}g/cm^3	0.755
μ/mm^{-1}	1.891
F(000)	1941.0
Crystal size/mm^3	0.06 × 0.05 × 0.05
Radiation	Mo Kα (λ = 0.71073)
2Θ range for data collection/°	3.63 to 56.558
Index ranges	-38 ≤ h ≤ 36, -38 ≤ k ≤ 38, -15 ≤ l ≤ 16
Reflections collected	137655
Independent reflections	4221 [R_{int} = 0.0584, R_{sigma} = 0.0161]
Data/restraints/parameters	4221/213/163
Goodness-of-fit on F^2	1.413
Final R indexes [I>=2σ (I)]	R_1 = 0.0871, wR_2 = 0.3072
Final R indexes [all data]	R_1 = 0.0925, wR_2 = 0.3172
Largest diff. peak/hole / e Å^{-3}	1.67/-2.23

Supercell Structure Determination and Refinement

For the supercell structure determination and refinement, the same dataset was used as for the average structure determination. Data reduction was conducted by using the software CrysAlisPro,[S2] this time including the diffuse scattering at *n* = half integers for the peak integration (Figure S4). Intensities integration was based on a primitive hexagonal unit cell, while absorption correction was conducted by a multiscan approach as implemented in CrysAlisPro.

Crystal structure solution and refinement was conducted by using the Olex2 software ver. 1.5.[S3] Structure solution in $P6_3/mmc$ was achieved by the program ShelXL[S5] used within the Olex2 user interface, revealing two Dy atoms of the metal cluster. The remaining atoms were assigned manually and a series of ShelXL restraints were used to drive the structure towards a reasonable molecular geometry (as derived from the average structure solution), which also agreed with the underlying electron density. The final refinement was conducted by the least square

algorithm of the ShelXL structure refinement program.[S5] All hydrogens were fixed in idealized positions and not localized directly from the electron density residues.

Solvent masking was conducted to neglect the influence, on the refinements, of minor residual densities that were not possible to assign with adequate confidence. The mask was created by the dedicated function of the software Olex2, which masked a void of 12628 Å^3 per unit cell (70.7%), containing 507 electrons belonging to possibly various kinds of solvent or modulator molecules.

Notably, the diffuse scattering leads to weak intensities and broad peak profiles in the *hk*-plane. This broadening limits the resolution of the Bragg-like reflections and complicates precise determination of atomic positions and thermal parameters. For this reason, the CIF file was not uploaded onto the CCDC. Despite these challenges, the refinement provides critical insights into the underlying structural motifs, including the unique Dy atom positions and occupancies within the clusters and the configurational relationship between adjacent clusters (Fig. S7). These key structural features align well with the proposed disorder model, demonstrating the robustness of the proposed structural framework despite the limited refinement quality. See Table S3 for final refinement parameters.

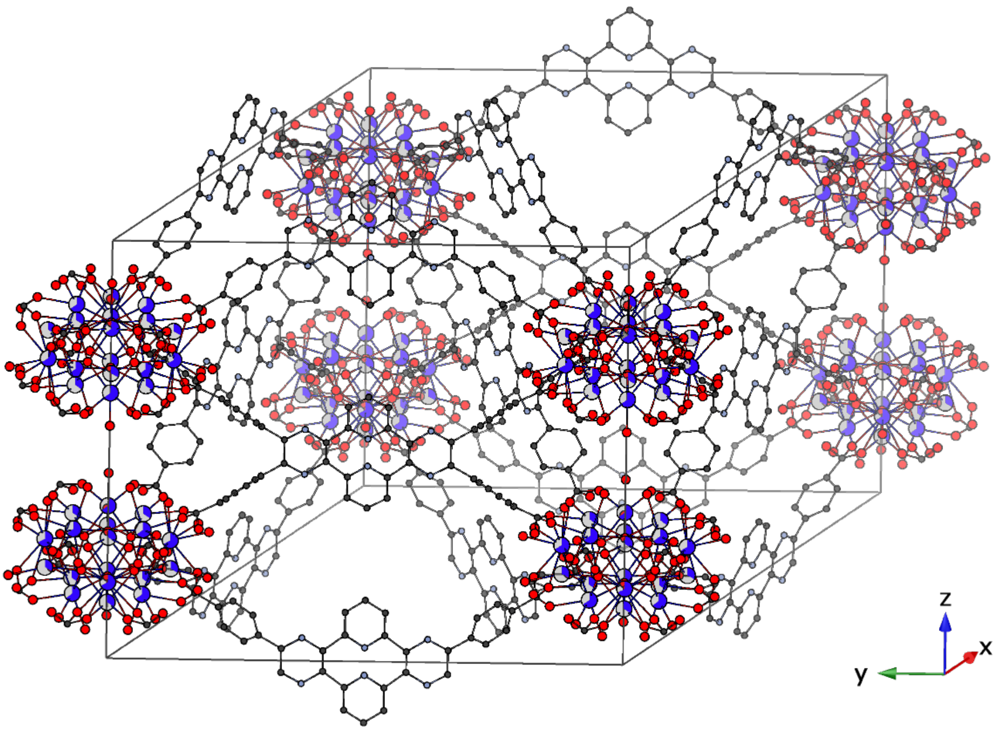

Supplementary Figure S7. Superstructure modelled by SCXRD data (entire unit cell), showing the partial occupancies of the Dy atoms as pie-charts. Colour scheme: blue/grey: Dy, light blue: N, dark grey: C, red: O. The hydrogen atoms are omitted for better clarity.

Supplementary Table S3 Crystal data and structure refinement.

Empirical formula	$C_{41}H_{19}Dy_3N_5O_{16}$
Formula weight	1323.94
Temperature/K	249.99(10)
Crystal system	hexagonal
Space group	P6/mmc
a/Å	28.6299(5)
b/Å	28.6299(5)
c/Å	25.1537(5)
α/°	90
β/°	90
γ/°	120
Volume/Å^3	17855.5(7)
Z	27
ρ_{calc}g/cm^3	0.739
μ/mm^{-1}	1.892
F(000)	3753.0
Crystal size/mm^3	0.06 × 0.05 × 0.05
Radiation	Mo Kα (λ = 0.71073)
2Θ range for data collection/°	4.31 to 56.558
Index ranges	-38 ≤ h ≤ 36, -38 ≤ k ≤ 38, -31 ≤ l ≤ 33
Reflections collected	284513
Independent reflections	8002 [R_{int} = 0.2068, R_{sigma} = 0.0384]
Data/restraints/parameters	8002/9/123
Goodness-of-fit on F^2	1.717
Final R indexes [I>=2σ (I)]	R_1 = 0.1278, wR_2 = 0.3875
Final R indexes [all data]	R_1 = 0.1535, wR_2 = 0.4264
Largest diff. peak/hole / e Å^{-3}	4.68/-3.62

1.2 Cluster Geometries

From the average structure of the node, the approach described in the text was used to obtain the two cluster geometries shown in Figure 1(a) in the main text.

Supplementary Table S4. Spin-up cluster geometry

Atom name	Fractional Coordinates		
C	-0.1748	0.0000	-0.1850
C	0.0000	0.1748	0.1850
C	0.0000	-0.1748	0.1850
C	0.0000	0.1748	-0.1850
C	-0.1748	0.0000	0.1850
C	-0.1748	-0.1748	0.1850
Dy	0.0809	0.1618	0.0000
O	0.0000	-0.0964	0.0000
O	-0.0335	0.1301	0.1960
O	-0.0307	-0.0614	-0.3530
O	0.1529	-0.0411	-0.1160
O	0.1529	0.1940	0.1160
C	0.1748	0.0000	0.1850
C	-0.1748	-0.1748	-0.1850
C	0.1748	0.1748	0.1850
C	0.1748	0.0000	-0.1850
C	0.0000	-0.1748	-0.1850
C	0.1748	0.1748	-0.1850
Dy	0.0899	0.0450	0.1544
O	-0.0964	0.0000	0.0000
O	-0.0335	-0.1636	-0.1960
O	-0.1940	-0.1529	-0.1160
O	-0.1940	-0.1529	0.1160
Dy	-0.0450	-0.0899	-0.1544
Dy	-0.1618	-0.0809	0.0000
O	-0.0964	-0.0964	0.0000
O	-0.1301	0.0335	0.1960
O	0.1636	0.0335	-0.1960
O	-0.0307	0.0307	0.3530
O	-0.1940	-0.0411	0.1160
Dy	0.0809	-0.0809	0.0000
O	0.0000	0.0964	0.0000
O	-0.1301	-0.1636	0.1960
O	0.1636	0.1301	0.1960
O	0.1636	0.1301	-0.1960

O	-0.0307	-0.0614	0.3530
O	0.0411	0.1940	-0.1160
O	0.1529	-0.0411	0.1160
O	-0.1940	-0.0411	-0.1160
Dy	-0.0450	-0.0899	0.1544
O	0.0000	0.0000	0.1076
O	-0.0335	0.1301	-0.1960
O	0.0614	0.0307	-0.3530
O	0.0411	-0.1529	0.1160
Dy	0.0899	0.0450	-0.1544
O	0.0000	0.0000	-0.1076
O	0.0964	0.0964	0.0000
O	0.1636	0.0335	0.1960
O	-0.0335	-0.1636	0.1960
O	-0.0307	0.0307	-0.3530
O	0.0614	0.0307	0.3530
O	0.1529	0.1940	-0.1160
Dy	-0.0450	0.0450	0.1544
O	0.0964	0.0000	0.0000
O	-0.1301	-0.1636	-0.1960
O	0.0411	0.1940	0.1160
Dy	-0.0450	0.0450	-0.1544
O	-0.1301	0.0335	-0.1960
O	0.0411	-0.1529	-0.1160

Supplementary Table S5. Spin-down cluster geometry

Atom name	Fractional Coordinates		
Dy	-0.0899	-0.0450	-0.1544
O	0.0000	0.0964	0.0000
Dy	0.1618	0.0809	0.0000
Dy	0.0450	0.0899	0.1544
Dy	0.0450	0.0899	-0.1544
Dy	0.0450	-0.0450	0.1544
O	0.0000	-0.0964	0.0000
O	0.0335	-0.1301	0.1960
O	0.0307	0.0614	0.3530
O	-0.1529	-0.1940	0.1160
O	-0.0411	-0.1940	-0.1160
Dy	-0.0809	-0.1618	0.0000
O	0.0000	0.0000	-0.1076
O	-0.1636	-0.1301	-0.1960
O	0.1301	0.1636	-0.1960
O	-0.0614	-0.0307	-0.3530
O	-0.0411	0.1529	-0.1160

C	0.1748	0.0000	0.1850
C	-0.1748	-0.1748	-0.1850
C	0.1748	0.1748	0.1850
C	0.0000	-0.1748	0.1850
C	0.0000	0.1748	-0.1850
C	-0.1748	-0.1748	0.1850
Dy	-0.0809	0.0809	0.0000
Dy	0.0450	-0.0450	-0.1544
O	0.0335	0.1636	-0.1960
O	-0.0964	-0.0964	0.0000
C	0.0000	-0.1748	-0.1850
C	0.1748	0.1748	-0.1850
C	0.1748	0.0000	-0.1850
O	0.0964	0.0964	0.0000
O	-0.1636	-0.0335	0.1960
O	-0.0964	0.0000	0.0000
O	0.1301	-0.0335	-0.1960
O	0.0307	-0.0307	-0.3530
O	0.0000	0.0000	0.1076
O	-0.1636	-0.1301	0.1960
O	-0.0614	-0.0307	0.3530
O	-0.1529	0.0411	0.1160
O	-0.1529	0.0411	-0.1160
O	-0.1636	-0.0335	-0.1960
O	-0.0411	-0.1940	0.1160
C	-0.1748	0.0000	-0.1850
C	0.0000	0.1748	0.1850
C	-0.1748	0.0000	0.1850
Dy	-0.0899	-0.0450	0.1544
O	0.0964	0.0000	0.0000
O	0.0335	-0.1301	-0.1960
O	0.1301	0.1636	0.1960
O	0.0335	0.1636	0.1960
O	0.0307	0.0614	-0.3530
O	0.1940	0.0411	0.1160
O	0.1940	0.0411	-0.1160
O	0.1301	-0.0335	0.1960
O	0.0307	-0.0307	0.3530
O	0.1940	0.1529	0.1160
O	0.1940	0.1529	-0.1160
O	-0.0411	0.1529	0.1160
O	-0.1529	-0.1940	-0.1160

Electrostatic treatment of the hexapole moment

In this work, we use the hexapole moment to describe the distribution of charge in the metal nodes of UoB-100. However, to understand how the hexapole moment translates to magnetic spins, we encode the hexapole moment using a mathematical object known as the octupole moment tensor. This tensor accounts for the position of the atoms, *k,* within the molecule, *j*, and their contribution to the charge distribution. The equation for the tensor, where some terms are zero by symmetry, is as follows:

$$[\Omega_i]_{\alpha\beta\gamma} = \frac{1}{2} \sum_{k \in \text{molecule } i} q_k \left(4r_{k\alpha} r_{k\beta} r_{k\gamma} - r^2 \big(r_{i\alpha}\, \delta_{\beta\gamma} + r_{i\beta}\, \delta_{\alpha\gamma} + r_{i\gamma}\, \delta_{\beta\gamma} \big) \right),$$

in which q_k is the charge on atom *k,* $r_{k\alpha}$ is the αth coordinate ($\alpha = x, y, z$) for the *k*th atom and $\delta_{\alpha\beta}$ is the Kronecker delta, which is equal to 1 if $\alpha = \beta$, and 0 otherwise.

This equation has the important property that if one inverts the coordinates, the sign of the hexapole moment changes. We can therefore factor express the moment as

$$\Omega_i = S_i \Omega_0,$$

where $S_i = \pm 1$, representing the two possible orientations of the molecule, and Ω_0 is the hexapole moment in one orientation.

Hexapole-hexapole interactions and relationship to magnetic spins

The interaction energy between two hexapoles (*i* and *j)* is governed by the rather complicated equation:

$$E_{ij} = \frac{1}{5r_{ij}^7 4\,\pi\,\epsilon_0} \Big(2\Omega_i \,\therefore\, \Omega_j + 42\big(n_{ij} \cdot \Omega_i\big) : \big(n_{ij} \cdot \Omega_j\big) + 126\big({n_{ij}}^{(2)} : \Omega_i\big) \cdot \big({n_{ij}}^{(2)} : \Omega_j\big) + 231\big({n_{ij}}^{(3)} \,\therefore\, \Omega_i\big)\big({n_{ij}}^{(3)} \,\therefore\, \Omega_j\big),$$

where $\cdot$, $:$ and $\therefore$ are the single, double and triple dot products and n_{ij}, ${n_{ij}}^{(2)}$, and ${n_{ij}}^{(3)}$ are the 1, 2 and 3 -rank unit vectors, as defined in Ref. S7. However, when simplifying the problem using the earlier definition $\Omega_i = S_i \Omega_0$, the interaction energy reduces to:

$$E_{ij} = J_{ij}\, S_i S_j,$$

where *J* is a constant depending on the distance, r_{ij}, between two molecules and their alignment:

$$J = \frac{1}{5r_{ij}^7 4\,\pi\,\epsilon_0} \Big(2\Omega_0 \,\therefore\, \Omega_0 + 42\big(n_{ij} \cdot \Omega_0\big) : \big(n_{ij} \cdot \Omega_0\big) + 126\big({n_{ij}}^{(2)} : \Omega_0\big) \cdot \big({n_{ij}}^{(2)} : \Omega_0\big) + 231\big({n_{ij}}^{(3)} \,\therefore\, \Omega_0\big)\big({n_{ij}}^{(3)} \,\therefore\, \Omega_0\big).$$

In this way, electrostatic hexapoles are equivalent to magnetic spins.

Estimating the size of the hexapole electrostatic interactions

To estimate the strength of the electrostatic 'hexapole' interactions between the clusters (Tables S4, S5), we assume the following charges (*q)*:

- Dysprosium atoms = +3
- Central oxygens = -2
- Formate oxygens = -0.5

Notably, this assumption provides an upper bound for the interaction strength because it neglects covalency, which would diminish these formal charges.

We calculate the nearest-neighbour interactions using:

$$E = \frac{N_{\mathrm{A}} q_{\mathrm{e}}^2}{4\pi\epsilon_0} \sum_{\{ij\}} \frac{q_i\, q_j}{|\mathbf{r_i} - \mathbf{r_j} + \boldsymbol{R}|},$$

where the sum over pairs $\{ij\}$ concerns atoms in different SBUs, separated by the inter-SBU vector **R**, $\mathbf{r}_i$ and $\mathbf{r}_j$ are the cluster coordinates of the *i*th and *j*th atoms (Tables S4, S5), q_{e} is the electron charge, ϵ_0 is the vacuum permittivity, N_{A} is Avogadro's number, and q_i and q_j are the formal charges of the *i*th and *j*th atoms respectively.

In this study, we consider both the (i) in-plane (*i.e.,* the *ab*-plane) and (ii) out-of-plane (*i.e.,* along the *c*-axis) interaction. We also note the following symmetry-imposed constraints: $E_{uu} = E_{dd}$ and $E_{ud} = E_{ud}$ for the up-up, down-down, up-down, and down-up pairs, respectively. For the in-plane interaction, we apply R = [*a*,0,0] to calculate $E_{uu} - E_{dd} = 0.0674$ kJmol^{-1}, and for the out-of-plane interaction, we apply R = [0,0,*c*] to calculate $E_{uu} - E_{dd} = 4.1110$ kJmol^{-1}.

The difference in size of these interactions is due to the difference in distance: the separation of the clusters along *c* is 2.27 times shorter than between those in the *ab*-plane, thus the hexapole-hexapole interaction ($\propto 1/r^7$) is two orders of magnitude stronger. Furthermore, the value for the in-plane interactions is an order of magnitude weaker than the steric factor calculated from DFT, which explains why the linker sterics overrule this interaction. In contrast, the value for the out-of-plane interactions is stronger than the steric interaction of the linkers, as well as of the same order of magnitude at room temperature, indicating the dominance of the hexapolar interactions along *c.*

Diffuse Scattering Measurement and Analysis

Experimental details

The single-crystal X-ray diffuse scattering measurement of a Dy-MOF crystal was collected on a RigakuXtaLAB Synergy diffractometer fitted with a HyPix detector. The dataset was collected under Cu radiation (λ = 1.5406 Å). Crystals were mounted on a MiTeGen loop using perfluoropolyether oil as a cryoprotectant. An exposure time of 10 s was used to detect diffuse temperatures at 100 K. The measurement involved a full ϕ-scan carried out in a single run. Raw data is available on request to the corresponding author.

3D Reciprocal Space Reconstruction

CrysAlisPro[S8] was used for indexing, determination and refinement of the orientation matrix. For the 3D reciprocal space reconstruction, we utilized the software Meerkat.[S9] As we observed a slight movement of the sample during the measurement, we utilized XDS[S10] to re-refine the orientation matrix after every 60° ϕ-rotation, which corresponds to 300 frames. The scattering data was then reconstructed on a three-dimensional grid defined by $-30 \leq h, k \leq +30$, $-30 \leq l \leq 30$ with voxel sizes of $\Delta h = \Delta k = \Delta l = 0.05$ reciprocal lattice units (r.l.u), resulting in an array of 1201 × 1201 × 1201 voxels. To improve statistics and cover missing parts of reciprocal space, the data were subsequently averaged for *6/mmm* Laue symmetry using a custom python script.

3D-ΔPDF extraction

Data treatment for the experimental 3D-ΔPDF generation consisted of Bragg peak removal and the subtraction of a constant background. Bragg peak removal was performed using a custom python script that punches a pre-defined area around the Bragg peak and interpolates the missing intensities. The Fast Fourier Transform (FFT) algorithm as implemented in Meerkat (REF) was used to obtain the 3D-ΔPDFs shown in Fig. 2 of the main text.

Computational Studies

Monte Carlo simulations

Overview

Monte Carlo (MC) simulations were carried out to generate configurations with representative diffuse scattering. Throughout, we assume that – since the correlation length is longer than our resolution allows us to determine – there is essentially long-range antiferrohexapolar order along *c*. There is, however, disorder within the plane governed by ferrohexapolar interactions, which was encoded in the Hamiltonian,

$$E_{MC} = J_{\perp} \sum_{\langle ij \rangle} \mathrm{S}_i \, \mathrm{S}_j$$

where $S_j = \pm 1$ represents which of the two states in Fig. 1(b) is present at site *j* in the lattice; $J_{\perp}$ is the coupling constant of nearest neighbours (denoted $\langle ij \rangle$). The two SBU orientations given in Tables S2.1 and S2.2 represent the atomic coordinates corresponding to the up and down spins. 'Simulated annealing' was used to generate several spin configurations which are decorated with the cluster geometries, for various values of the $J_{\perp}$ parameter. We then calculated the diffuse scattering from these simulations. The free $J_{\perp}$ parameter was fitted to a representative region of reciprocal space, giving an indication of the strength of the correlations.

Details of the simulations

Simulations of the nnHAF model were carried out using the metropolis algorithm.[S11] The MC simulation was carried out using a custom code related to that used in Ref. S12. All simulations were carried out on 24×24 supercells. To reach the ground state we used the approach of simulated annealing, cooling from T = 15 to 5 $J_{\perp}$ on a logarithmically spaced grid of 23 points. For each temperature, the algorithm measures the decorrelation time n_d, equilibrates for $10n_\mathrm{d}$, and progresses to measure 80 samples with $2n_\mathrm{d}$ moves per sample. In this way, we ensure ergodicity in the simulations.

Calculation of diffuse scattering

Calculating the diffuse scattering required decorating the 24×24×1 supercells with the cluster geometries presented in Tables S2 and S3, and using the software SCATTY (which exploits fast-Fourier transform) to calculate various planes.[S13] To include the antiferrohexapolar order along the *c* direction, it was necessary to extend the 24 $J_{\perp}/T$ 24×1 supercells to 24×24×2, using the rule $S(X,Y,Z+1) = -S(X,Y,Z)$. SCATTY was used to calculate the *hk*0.5 plane, with no Lanczos resampling. The maximum in-plane resolution of the simulations was given by the size of the supercell: 1/24 r.l.u.

However, the 24×24×2 supercells did not give sufficient resolution along *c* to simulate the *h*0*l* plane. To do so, we therefore used the convolution theorem, noting the periodicity along the *c*-axis to extend the resolution arbitrarily in this direction. The Fourier transform of the periodic lattice along the *c*-direction is a set of planes perpendicular to *c*, which were multiplied by the simulated *h*0*l* planes to give the data plotted in Figure 2(a) of the main text. The width of these planes is ultimately arbitrary as they are resolution limited in the experiment, but a width was chosen that makes them visible.

Parameterization of the model

The Hamiltonian was parameterized by fitting to the scattering data. As noted in the text and by Schmidt and Neder,[S14] in the case of disorder between two orientations of a molecule, only one Brillouin zone is required to encode all of the correlations present. For this reason, a representative region of reciprocal space was chosen to parametrize the model, bounded by [7 0 0.5] ± [0.5 0.5 0]

Since the scattering considered is localized in the *hk*0.5 plane, to remove the background, a region 0.05 r.l.u. above and below the plane was subtracted to give the experimental diffuse scattering in this region, shown in Figure S8(a). By simulated annealing, a number of different values of $J_{\perp}/T$ were sampled, and the corresponding diffuse scattering (which becomes sharper at high-coupling and diffuse at low-coupling) was plotted. In this way, we could fit the experimental sharpness of this feature. Normalization of these two datasets was achieved by matching the maximum intensity within the region of reciprocal space. The resulting residual is shown in Figure S8(b), with a goodness-of-fit as a function of our $J_{\perp}/T$ parameter,

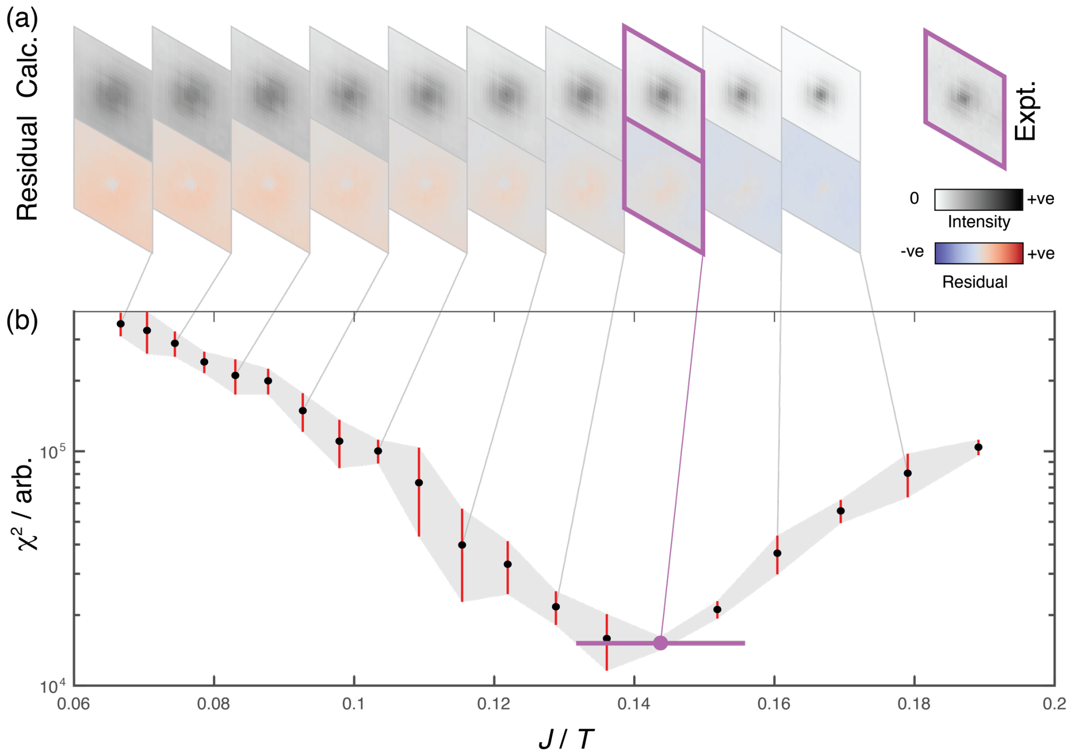

Supplementary Figure S8: (a) Calculation of the reciprocal-space region discussed in the text, which is normalized to have the same maximum intensity. The residual of these calculated regions is given below each panel for comparison. The experimental region is shown on the right, highlighted by the purple line. (b) The overall χ^2 of the data shown on a log-plot, with error-bars (red) representing 2σ-uncertainty in the value of χ^2. The corresponding uncertainty in the fitted value of $J_{\perp}/T$ is shown as the purple bar. Gray lines show the corresponding plots in parts (a) and (b)

$$\chi^2 = \sum_{h,k} (I^{\mathrm{exp}}\,(h, k, 0.5) - I^{\mathrm{calc}}(h, k, 0.5))^2$$

This whole procedure was repeated 4 times, and a distribution of χ^2 values across these samples gave the mean and 2σ-uncertainty plotted in Figure S7(b). These uncertainties bound a feasible region (gray) for the χ -dependence of $J_\perp/T$. The uncertainty (shown as a purple bar) on the fit parameter is estimated as the range of parameters for which the fit is not *significantly* better than the best fit. With this approach, we extract the best fit of the model-parameter as J = 0.144(12) T_{eff}.

Calculation of 3D-ΔPDF

We calculated the full volume of reciprocal space from the best-fit configurations in the same way as for the other scattering data, using SCATTY.[S13] We used a max-*hkl* of 20 r.l.u in each direction, and a 401×401×81 grid of reciprocal lattice points, giving us the maximum resolution of the 24×24×2 supercells in the *c*-direction. In this way, we obtain the data plotted in Figure 2(d). To broaden the simulation to match the real-space experimental resolution, a gaussian convolution of a width of 0.0833 r.l.u is applied, as matched by eye to give the best-agreement with experiment. Rather than using the multiplication approach used to calculate the diffuse scattering, we can directly apply the periodicity in the *c*-direction to obtain the 3D-ΔPDF presented in Figure 2(d) of the main text.

Quantum Mechanical Calculations

Our analysis up to this point has intentionally ignored the presence of the linker, instead inferring the correlations between cluster orientations. The objective of this next part is to understand how the linker may relax locally between a decoration of neighboring SBUs, given their relative orientation. Linkers connect 4 neighboring clusters, hence there are two possible neighboring SBU orientations that respect the long-range order along the *c*-direction, which we will refer to as ferro- and antiferrohexapolar configurations, as shown in Figure 4(b-c) in the main text. In the former case, the SBUs are in the same orientation on either side of the linker, while in the latter case, they are in a different orientation.

Geometry optimization was carried out on the charged linker, $[C_{39}H_{19}N_7O_8]^{4-}$, constraining the oxygen positions of the carboxylate groups connected to the node by their crystallographic positions, given in Tables S2 and S3. The remaining molecular confirmation was allowed to relax, starting from a rough estimate of the atomic positions, which are given in Tables S4 and S5. This rough estimate was generated by assuming the molecule lies entirely in the plane of the carboxylate carbon atoms, apart from the terminal benzene rings which are perpendicular to this plane [See iteration 1 in Figure S9] and the only difference between the two starting configurations are the oxygen positions. Since the relative orientations of the SBUs are different in the ferro- and antiferrohexapolar configurations, the carboxylate oxygen positions will also be different, leading to distinct optimal geometries and final energies.

DFT calculations were carried out using the software package ORCA,[S15] using the PBE functional,[S15] and the def2-svp basis set.[S16] By default, the TightSCF convergence of the SCF cycles is used,[S17] and the NormalOpt criterial are used for the geometry optimization. Two symmetry constraints were used: for the antiferro configuration, the starting model had C_2 symmetry, while for the ferro configuration, we started with the point group C_s.

The resulting trajectories, shown in Figure S9, show that the linker connecting antiferrohexapolar SBUs bends into an S-shape, but maintains the orientation along *c* guaranteed by the symmetry. For the ferrohexapolar arrangement, on the other hand, the linker relaxes much more dramatically, slowly twisting the linker to lie in the plane through bending in a U-shape. The final coordinates are given in Tables S4 and S5.

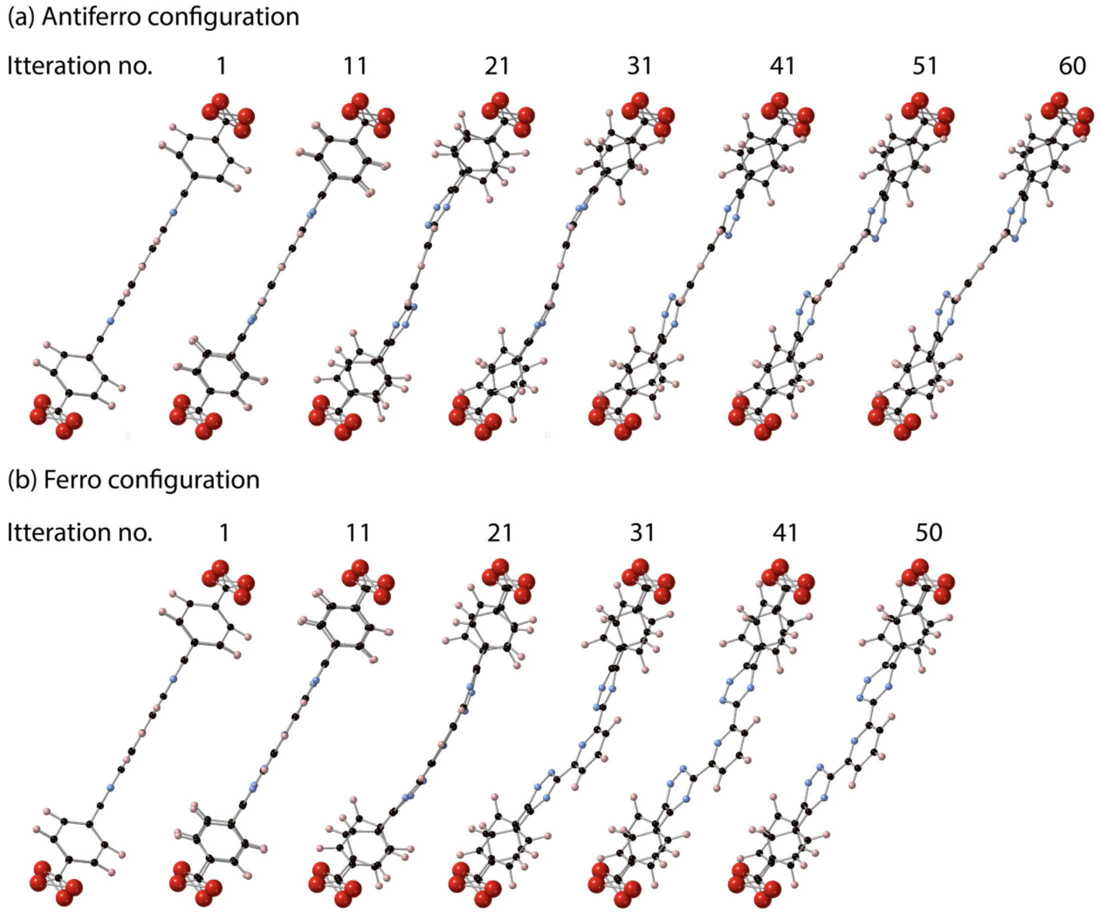

Supplementary Figure S9. Trajectories of DFT relaxation, viewed down the *c*-axis. Linker between (a) antiferrohexapolar oriented SBUs, where the oxygen positions induce a slight S-shaped bend into the linker and (b) ferrohexapolar oriented SBUs, where the linker bends in the middle before twisting towards the plane.

Supplementary Table S6: Antiferrohexapolar linker coordinates before and after relaxation. Starred atoms are constrained by their neighboring SBU

Atom type	Initial cartesian coordinates / Å			Final cartesian coordinates / Å		
C	4.659498	8.070487	-4.22666	4.573987	8.009104	-4.42881
C	4.084951	7.075342	-3.47337	3.72294	6.893043	-3.78831
C	2.89373	5.012087	-1.80266	2.750939	4.847596	-2.10573
C	2.361264	4.08983	-1.04947	2.42482	3.801522	-1.12917
C	2.333225	4.041265	0.52288	2.287837	4.031182	0.284789
C	1.183949	2.050659	0.588417	1.4336	1.909154	0.4144
C	2.935811	5.084974	1.342036	2.838645	5.136399	1.07855
C	4.071	7.051179	2.914276	3.990028	6.981926	2.861302
C	4.659498	8.070487	3.700503	4.597811	8.050054	3.777844
C	0.581363	1.00695	1.341993	0.687008	0.921711	1.217302
C	0.567458	0.982865	2.914308	0.70899	0.981015	2.630393
C	0.006816	0.011805	3.634592	-0.0061	0.010606	3.341784
C	-0.59572	-1.03183	2.946852	-0.72099	-0.95995	2.630384
C	-0.56773	-0.98334	1.27651	-0.69883	-0.90073	1.217293
C	-1.17032	-2.02705	0.522891	-1.44296	-1.88982	0.414106
C	-2.3335	-4.04174	0.588374	-2.28961	-4.01495	0.284206
C	-2.3335	-4.04174	-1.0165	-2.42653	-3.78576	-1.1298
C	-2.92218	-5.06136	-1.80265	-2.74928	-4.83293	-2.10644
C	-2.93627	-5.08577	1.309054	-2.83658	-5.12234	1.077566
C	-4.08536	-7.07605	-3.40791	-3.71701	-6.88086	-3.78839
C	-4.05737	-7.02757	2.946852	-3.98365	-6.97128	2.859905
C	-4.65991	-8.0712	3.667532	-4.5907	-8.03962	3.776579
C	-4.64582	-8.0468	-4.19408	-4.5675	-7.99756	-4.42859
C	4.912631	5.910558	-3.05556	4.024201	5.544287	-4.06031
C	4.316998	4.878891	-2.22058	3.535552	4.525277	-3.24121
C	2.661637	7.208459	-3.05556	2.830406	7.198746	-2.7447
C	2.066004	6.176792	-2.22058	2.357559	6.192126	-1.89898
C	4.34522	4.927773	1.735256	4.090619	5.721163	0.777761
C	4.912814	5.910875	2.521377	4.668108	6.610456	1.685052
C	2.094226	6.225674	1.735256	2.16554	5.496366	2.272989
C	2.66182	7.208776	2.521377	2.72405	6.436847	3.135489
C	-2.08752	-6.21402	-2.20387	-2.35338	-6.1766	-1.89912
C	-2.66906	-7.22128	-3.0067	-2.82424	-7.18456	-2.74449
C	-4.33848	-4.91614	-2.20387	-3.53414	-4.51246	-3.24229
C	-4.92002	-5.9234	-3.0067	-4.02045	-5.53268	-4.06118
C	-2.65155	-7.19094	2.539284	-2.71972	-6.4219	3.135131
C	-2.09109	-6.2202	1.724554	-2.16355	-5.47968	2.272885
C	-4.9025	-5.89306	2.539284	-4.66138	-6.60276	1.682503

C	-4.34205	-4.92232	1.724554	-4.08591	-5.71204	0.775437
N	1.74459	3.021719	-1.79475	2.320653	2.530908	-1.58387
N	1.184635	2.051847	-1.06059	1.887013	1.550258	-0.81193
N	1.731005	2.998189	1.269336	1.67798	3.079892	0.99989
N	-0.00334	-0.00578	0.535196	-0.00613	0.010655	0.52664
N	-1.16419	-2.01643	-1.06054	-1.8966	-1.53246	-0.81241
N	-1.76508	-3.05721	-1.82686	-2.3263	-2.51483	-1.58458
N	-1.7515	-3.03368	1.269368	-1.68375	-3.06131	0.999608
O*	-5.77252	-8.33675	3.561904	-5.77253	-8.33675	3.561904
O*	-3.7895	-8.6021	4.568504	-3.7895	-8.6021	4.568504
O*	-4.33358	-9.16753	-4.08826	-4.33358	-9.16753	-4.08826
O*	-5.55489	-7.58285	-5.09486	-5.55489	-7.58285	-5.09486
O*	3.796129	8.613593	4.568504	3.796129	8.613592	4.568504
O*	5.779158	8.34824	3.561904	5.779159	8.348239	3.561904
O*	4.340209	9.179017	-4.08826	4.340208	9.179017	-4.08826
O*	5.561526	7.59434	-5.09486	5.561524	7.59434	-5.09486
H	5.877343	5.354315	-3.05556	4.73872	5.363785	-4.87962
H	5.281709	4.322648	-2.22058	3.802897	3.469383	-3.40057
H	1.696925	7.764702	-3.05556	2.612046	8.266285	-2.57968
H	1.101292	6.733035	-2.22058	1.719576	6.442574	-1.0371
H	5.309931	4.37153	1.735256	4.61408	5.44273	-0.14976
H	5.877526	5.354632	2.521377	5.659369	7.063637	1.527916
H	1.129514	6.781917	1.735256	1.1975	5.018781	2.48829
H	1.697108	7.765019	2.521377	2.217571	6.804218	4.042499
H	-1.12282	-6.77025	-2.20387	-1.71537	-6.42544	-1.03677
H	-1.70436	-7.77752	-3.0067	-2.60408	-8.25156	-2.57838
H	-5.30318	-4.3599	-2.20387	-3.80346	-3.45713	-3.40199
H	-5.88473	-5.36716	-3.0067	-4.73512	-5.35451	-4.88088
H	-1.68684	-7.74718	2.539284	-2.21266	-6.78677	4.04282
H	-1.12638	-6.77643	1.724554	-1.19753	-4.99851	2.489287
H	-5.86721	-5.33682	2.539284	-5.65113	-7.05887	1.524444
H	-5.30675	-4.36608	1.724554	-4.60947	-5.43607	-0.15276
H	0.810431	1.403708	3.489781	1.28773	1.775274	3.121972
H	-0.01134	-0.01965	4.506477	-0.00601	0.01039	4.443945
H	-0.85722	-1.48475	3.150829	-1.29916	-1.75462	3.122002

Supplementary Table S7: Ferrohexapolar linker coordinates before and after relaxation. Starred atoms are constrained by their neighboring SBU

Atom type	Initial cartesian coordinates / Å			Final cartesian coordinates / Å		
C	4.656181	8.064742	-3.96349	4.605397	8.007203	-4.09967
C	4.081634	7.069598	-3.21019	4.08071	6.739437	-3.40944
C	2.890413	5.006343	-1.53948	3.015773	4.695629	-1.80789
C	2.357948	4.084085	-0.78629	2.437458	3.76797	-0.83758
C	2.329909	4.03552	0.786056	2.850817	3.727951	0.537311
C	1.180632	2.044914	0.851593	1.7691	1.715525	0.65531
C	2.932495	5.07923	1.605212	3.403829	4.848664	1.303394
C	4.067683	7.045434	3.177452	4.276304	6.925648	2.990339
C	4.656181	8.064742	3.963679	4.764407	8.009869	3.965176
C	0.578046	1.001205	1.605169	1.533937	0.444355	1.370383
C	0.564141	0.977121	3.177484	2.115815	0.178388	2.632562
C	0.003499	0.006061	3.897769	1.800472	-1.03692	3.255062
C	-0.59904	-1.03757	3.210028	0.907799	-1.91866	2.630893
C	-0.57105	-0.98908	1.539686	0.386737	-1.54709	1.368672
C	-1.17363	-2.03279	0.786067	-0.59465	-2.38747	0.651936
C	-2.33682	-4.04748	0.85155	-1.80611	-4.32504	0.541148
C	-2.33682	-4.04748	-0.75332	-2.04178	-3.9943	-0.83645
C	-2.9255	-5.06711	-1.53947	-2.55895	-4.96125	-1.80357
C	-2.93958	-5.09151	1.57223	-2.50425	-5.35894	1.311411
C	-4.08868	-7.0818	-3.14474	-3.79979	-6.9047	-3.40325
C	-4.06069	-7.03331	3.210028	-3.86365	-7.15556	2.998116
C	-4.66323	-8.07694	3.930708	-4.55538	-8.1279	3.967685
C	-4.64914	-8.05254	-3.9309	-4.63325	-7.99206	-4.09793
C	4.909314	5.904814	-2.79238	4.911962	6.04404	-2.51528
C	4.313681	4.873146	-1.9574	4.385887	5.036974	-1.70923
C	2.65832	7.202714	-2.79238	2.741542	6.340367	-3.57296
C	2.062687	6.171047	-1.9574	2.206784	5.315052	-2.78889
C	4.341903	4.922028	1.998432	4.199779	4.558784	2.436709
C	4.909497	5.905131	2.784553	4.646045	5.592412	3.252534
C	2.090909	6.219929	1.998432	3.0602	6.192501	1.01613
C	2.658503	7.203031	2.784553	3.47843	7.216313	1.871384
C	-2.09084	-6.21976	-1.94069	-2.17824	-6.32035	-1.697
C	-2.67238	-7.22703	-2.74352	-2.78871	-7.27926	-2.50229
C	-4.34179	-4.92188	-1.94069	-3.49321	-4.56882	-2.79034
C	-4.92334	-5.92915	-2.74352	-4.11587	-5.54444	-3.57309
C	-2.65486	-7.19668	2.80246	-2.52654	-6.80289	3.263835
C	-2.0944	-6.22594	1.987731	-1.85645	-5.89832	2.447964
C	-4.90582	-5.8988	2.80246	-4.51428	-6.61266	1.877817
C	-4.34536	-4.92806	1.987731	-3.83822	-5.73737	1.022572
N	1.741274	3.015974	-1.53157	1.441232	2.946263	-1.22802

N	1.181318	2.046103	-0.79741	1.027101	1.960569	-0.4564
N	1.727689	2.992445	1.532512	2.610401	2.593737	1.205434
N	-0.00666	-0.01153	0.798372	0.718746	-0.41171	0.751066
N	-1.1675	-2.02218	-0.79737	-1.16757	-1.87263	-0.46738
N	-1.7684	-3.06296	-1.56369	-1.81682	-2.72505	-1.2357
N	-1.75481	-3.03943	1.532544	-0.94308	-3.54978	1.208156
O*	-5.77584	-8.3425	3.82508	-5.77584	-8.3425	3.82508
O*	-3.79281	-8.60785	4.83168	-3.79281	-8.60785	4.83168
O*	-4.33689	-9.17327	-3.82508	-4.33689	-9.17327	-3.82508
O*	-5.55821	-7.5886	-4.83168	-5.55821	-7.5886	-4.83168
O*	5.558209	7.588595	4.83168	5.558209	7.588595	4.83168
O*	4.336892	9.173272	3.82508	4.336892	9.173272	3.82508
O*	5.775841	8.342495	-3.82508	5.775841	8.342495	-3.82508
O*	3.792812	8.607848	-4.83168	3.792812	8.607848	-4.83168
H	5.874026	5.34857	-2.79238	5.954698	6.393333	-2.44719
H	5.278393	4.316903	-1.9574	5.014082	4.528869	-0.96037
H	1.693609	7.758958	-2.79238	2.149024	6.920457	-4.29933
H	1.097976	6.72729	-1.9574	1.150524	5.011361	-2.86001
H	5.306615	4.365785	1.998432	4.450635	3.507078	2.643958
H	5.874209	5.348887	2.784553	5.290314	5.445271	4.133752
H	1.126197	6.776172	1.998432	2.439503	6.418475	0.135775
H	1.693792	7.759275	2.784553	3.205845	8.271869	1.711927
H	-1.12613	-6.776	-1.94069	-1.42959	-6.61138	-0.94309
H	-1.70768	-7.78327	-2.74352	-2.5757	-8.3579	-2.43116
H	-5.3065	-4.36564	-1.94069	-3.75114	-3.5009	-2.86737
H	-5.88804	-5.37291	-2.74352	-4.90958	-5.31939	-4.30431
H	-1.69016	-7.75292	2.80246	-2.07634	-7.28551	4.145719
H	-1.1297	-6.78218	1.987731	-0.82149	-5.58607	2.655745
H	-5.87052	-5.34256	2.80246	-5.56316	-6.90873	1.71628
H	-5.31007	-4.37182	1.987731	-4.34345	-5.31689	0.139758
H	0.807115	1.397964	3.752957	2.777215	0.92836	3.088732
H	-0.01466	-0.02539	4.769653	2.233243	-1.28697	4.237657
H	-0.86054	-1.4905	3.414005	0.589871	-2.86755	3.085115

References

[S1] G. K. Angeli, et al., *ACS Appl. Mater. Interfaces*, **9**, 44560-44566 (2017).

[S2] Rigaku Oxford Diffraction, 2023, CrysAlisPro Software System, Version 1.171.43.90, Oxford, UK OlexSys Ltd. 2004 – 2024;

[S3] O. V.; Dolomanov, L. J. Bourhis, R. J. Gildea, J. A. K. Howard, H. Puschmann, OLEX2: A Complete Structure Solution, Refinement and Analysis Program. *J Appl Crystallogr*, **42**, 339–341 (2009). https://doi.org/10.1107/S0021889808042726

[S4] *SHELXT* – Integrated space-group and crystal-structure determination, *Acta Crystallographica A*, **71**, 3–8 (2015).

[S5] G. M. Sheldrick, IUCr. Crystal Structure Refinement with SHELXL. *Acta Crystallographica Section C*, **71**, 3–8 (2015).

[S6] J.P. Vizuet, M.L. Mortensen, A.L. Lewis, M.A. Wunch, H.R. Firouzi, G.T. McCandless, K.J. Balkus Jr., Fluoro-Bridged Clusters in Rare-Earth Metal–Organic Frameworks, *J. Am. Chem. Soc.*, **143**, 17995–18000 (2021).

[S7] T. Ichiye and M-L. Tan, *J. Chem. Phys.* **124**, 134504 (2006).

[S8] Agilent (2014). CrysAlis PRO. Agilent Technologies Ltd, Yarnton, Oxfordshire, England.

[S9] A. Simonov, (2020). *Meerkat.* Version 0.3.7. A program for reciprocal space reconstruction. https://github.com/aglie/meerkat.

[S10] Kabsch, W. XDS. Acta Cryst. D**66**, 125-132 (2010).

[S11] N. Metropolis, A. W. Rosenbluth, M. N. Rosenbluth, A. H. Teller, and E. Teller, *J. Chem. Phys.* **21**, 1087 (1953).

[S12] J.A.M. Paddison, J. R. Stewart, P. Manuel, P. Courtois, G. J. McIntyre, B. D. Rainford, and A. L. Goodwin, *Phys. Rev. Lett.* **110**, 267207 (2013).

[S13] J.A.M. Paddison, Ultrafast calculation of diffuse scattering from atomistic models, Acta Cryst A **75**, 14-24 (2019).

[S14] E. Schmidt, R.B. Neder, *Acta Cryst. A* **73**, 231-237 (2017).

[S15] F. Neese, *The ORCA program system Wiley Interdiscip. Rev.: Comput. Mol. Sci.*, **2**, 73–78 (2012).

[S16] P. Perdew, K. Burke, M. Ernzerhof, Generalized Gradient Approximation Made Simple, Phys Rev Lett. 77 (1996) 3865–3868. https://doi.org/10.1103/PhysRevLett.77.3865.

[S17] F. Weigend, Accurate Coulomb-fitting basis sets for H to Rn. *Phys. Chem. Chem. Phys.*, 2006, 8, 1057-1065
